# 6-Methyl-2-oxo-*N*-(quinolin-6-yl)-2*H*-chromene-3-carboxamide: crystal structure and Hirshfeld surface analysis

**DOI:** 10.1107/S2056989016011026

**Published:** 2016-07-12

**Authors:** Lígia R. Gomes, John Nicolson Low, André Fonseca, Maria João Matos, Fernanda Borges

**Affiliations:** a FP–ENAS–Faculdade de Ciências de Saúde, Escola Superior de Saúde da UFP, Universidade Fernando Pessoa, Rua Carlos da Maia, 296, P-4200-150 Porto, Portugal; b REQUIMTE, Departamento de Química e Bioquímica, Faculdade de Ciências da Universidade do Porto, Rua do Campo Alegre, 687, P-4169-007 Porto, Portugal; c Department of Chemistry, University of Aberdeen, Meston Walk, Old Aberdeen AB24 3UE, Scotland; d CIQUP/Departamento de Química e Bioquímica, Faculdade de Ciências, Universidade do Porto, 4169-007 Porto, Portugal

**Keywords:** crystal structure, coumarin, carboxamide, Hirshfeld surface analysis

## Abstract

The title coumarin derivative, C_20_H_14_N_2_O_3_, displays intra­molecular N—H⋯O and weak C—H⋯O hydrogen bonds, which probably contribute to the approximate planarity of the mol­ecule [dihedral angle between the coumarin and quinoline ring systems = 6.08 (6)°]. The supra­molecular structures feature C—H⋯O hydrogen bonds and π–π inter­actions, as confirmed by Hirshfeld surface analyses.

## Abstract 2

The 6-methyl-2-oxo-*N*-(quinolin-6-yl)-2*H*-chromene-3-carboxamide coumarin derivative displays intra­molecular N—H⋯O and weak C—H⋯O hydrogen bonds, which probably contribute to the approximate planarity of the mol­ecule [dihedral angle between the coumarin and quinoline ring systems = 6.08 (6)°]. The supra­molecular structures feature C—H⋯O hydrogen bonds and π–π inter­actions, as confirmed by Hirshfeld surface analyses.

## Chemical context   

Coumarin and its derivatives are widely recognized by their unique biological properties (Matos *et al.*, 2014 ▸; Vazquez-Rodriguez *et al.*, 2013 ▸; Chimenti *et al.*, 2010 ▸). Our work in this area has shown that coumarin is a valid scaffold for the development of new drugs for aging related diseases, specifically within the class of mono­amino oxidase B inhibitors (Matos *et al.*, 2009 ▸). On the other hand, quinoline is a nitro­gen heterocycle also often used in drug-discovery programs due to its remarkable biological properties, some of them related to neurodegenerative diseases (Sridharan *et al.*, 2011 ▸), for instance, as γ-secretase and acetyl­cholinesterase inhibitors (Camps *et al.*, 2009 ▸). As part of our ongoing studies in this area (Gomes *et al.*, 2016 ▸), we describe the synthesis and crystal structure of the title coumarin–quinoline hybrid, 6-methyl-2-oxo-*N*-(quinolin-6-yl)-2*H*-chromene-3-carboxamide, (1) (see Scheme).
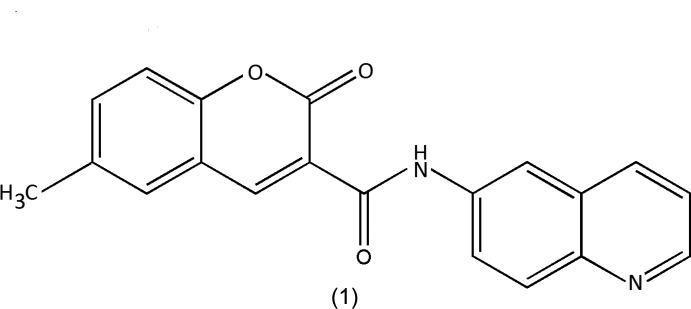


## Structural commentary   

**Figure 1:**
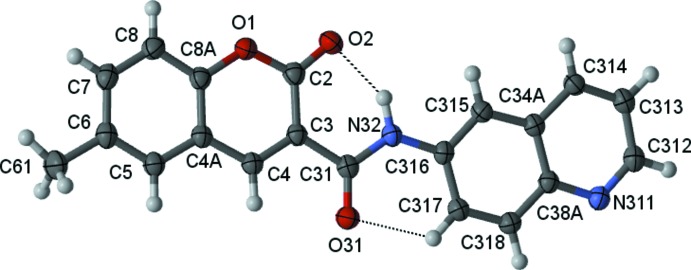
A view of the asymmetric unit of (1), showing the atom-numbering scheme. Displacement ellipsoids are drawn at the 70% probability level.

Fig. 1 ▸ shows an ellipsoid plot of the mol­ecular structure of (1). An inspection of the bond lengths shows that there is a slight asymmetry of the electronic distribution around the coumarin ring: the C3—C4 [1.3609 (15) Å] and C3—C2 [1.4600 (18) Å)] bond lengths are shorter and longer, respectively, than those expected for a C_ar_—C_ar_ bond, suggesting that the electronic density is rather located near the C3—C4 bond at the pyrone ring, as occurs in other coumarin-3-carboxamide derivatives (Gomes *et al.*, 2016 ▸). Also, the C3—C31 bond length [1.5075 (18) Å] is similar to the mean value displayed by other coumarin-3-carboxamide derivatives previously characterized (Gomes *et al.*, 2016 ▸) and is of the same order as a C*sp* ^3^—C*sp* ^3^ bond.

The C—N rotamer of the amide group governs the conformation of the mol­ecule: the −*anti* orientation where the N atom is −*cis* positioned with respect to the oxo O atom of the coumarin system allows the establishment of an intra­molecular N32—H32⋯O2 hydrogen bond between the amino group of the carboxamide and the oxo group of the coumarin system, and of a weak intra­molecular C317—H317⋯O31 hydrogen bond that connects the quinoline ring with the O atom of the carboxamide group (Table 1 ▸). Both these inter­actions form *S*(6) rings and connect the spacer carboxamide group with the heteroaromatic rings, probably constraining the rotation/bending of those rings with respect to the plane formed by the amide atoms. In fact, the mol­ecule is roughly planar, as may be evaluated by the set of values for the dihedral angles which are less than 7° (Table 2 ▸).

## Supra­molecular features   

**Figure 2:**
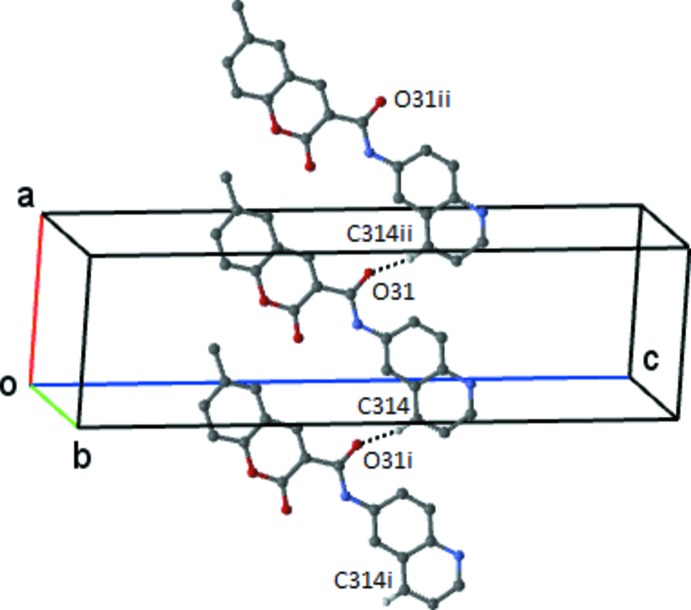
The simple *C*4 chain in compound (1) formed by the weak C314—H314⋯O3^i^ hydrogen bond. This chain extends by unit translation along the *a* axis. H atoms not involved in the hydrogen bonding have been omitted. [Symmetry codes: (i) *x* − 1, *y*, *z*; (ii) *x* + 1,*y*, *z*.]

In the crystal of (1), mol­ecules are linked by a weak C314—H314⋯O31^i^ hydrogen bond to form a *C*(8) chain, which runs parallel to the *a* axis (Fig. 2 ▸ and Table 1 ▸). There are several π–π contacts that will be described below.

## Hirshfeld surface analyses   

**Figure 3:**
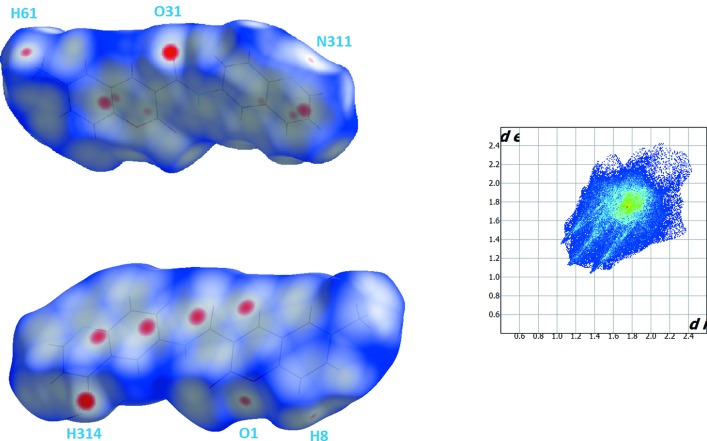
Views of the Hirshfeld surface mapped over *d*_norm_ (left) and fingerprint plot (right, FP) for (1). The highlighted red spots on the top face of the surfaces indicate contact points with the atoms participating in the inter­molecular C—H⋯O inter­actions, whereas those on the middle of the surface corresponds to C⋯C contacts consequent of the π–π stacking. The C⋯C contacts contribute to the higher frequency of the pixels at *d*_e_/*d*_i_ at 1.8° on the FP plot (yellow spot). The FP plot displays two light-blue spikes (external ends corresponding to C⋯ H contacts).

The Hirshfeld surfaces and two-dimensional fingerprint (FP) plots (Rohl *et al.*, 2008 ▸) were generated using *Crystal Explorer* (Wolff *et al.*, 2012 ▸). Compound (1) has three O atoms and an N atom that can potentially act as acceptors for hydrogen bonds, but one of the lone pairs of the oxo O atoms of the coumarin nucleus and of the amide moiety are involved in the establishment of intra­molecular hydrogen bonds, as discussed above. As such, they contribute to the electronic density of the pro-mol­ecule in the calculation of the Hirshfeld surface, leaving only the remaining pairs available for participation in the supra­molecular structure formation. The surface mapped over *d*_norm_ displays several red spots that correspond to areas of close contacts between the surface and the neighbouring environment, and the FP plot is presented in Fig. 3 ▸.

The contributions from various contacts, listed in Table 3 ▸, were selected by partial analysis of the FP plot. Taking out the H⋯H contacts on the surface that are inherent to organic mol­ecules, the most significant contacts can be divided in three groups: (i) H⋯O/O⋯H together with H⋯N/N⋯H that correspond to weak C—H⋯O/N inter­molecular inter­actions (24.5%); (ii) C⋯C and N⋯C/C⋯N contacts that are related with π–π stacking (17.9%): (iii) H⋯C/C⋯H contacts (14.3%).

The H⋯N/O contacts appear as three highlighted red spots on the top and bottom edges of the surface which form pairs of spots of comlementary size, indicating the contact points of the labelled atoms participating in the C–H⋯N/O inter­actions (Fig. 3 ▸). The strongest spots correspond to oxo atom O31 of the carboxamide acceptor and donor atom H314, which forms the C314—H314⋯O31^i^ hydrogen bond (Table 1 ▸), and the other spots correspond to very weak hydrogen-bond contacts, one involving pyrone atom O1 and a H atom of the methyl group (C61—H61*B*⋯O1^ii^; Table 1 ▸), and the other appearing perpendicular to the quinoline N atom indicating a very weak C8—H8⋯N311^ii^ contact (Table 1 ▸). In spite of the weakness of these contacts, their relative strength is reflected in the FP plots where the pair of sharp spikes pointing to south-west is highlighted in light blue.

**Figure 4:**
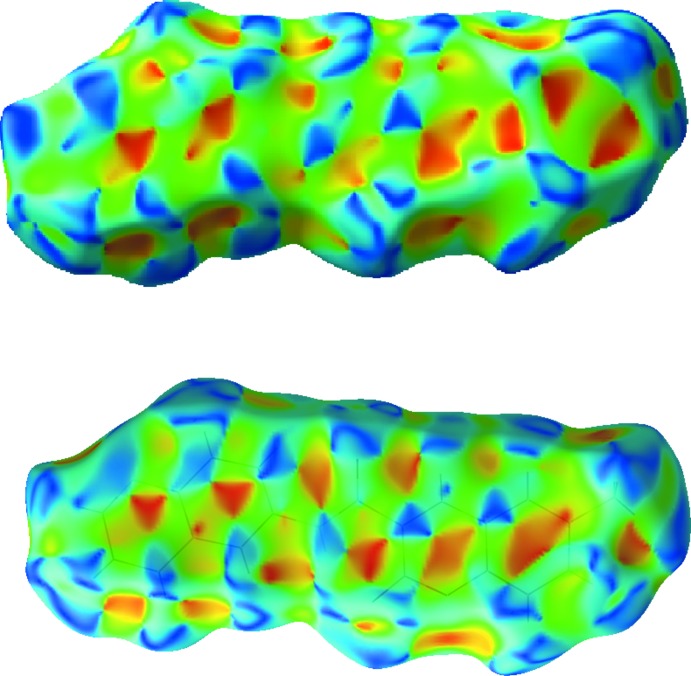
Shape index plots showing the inter­actions arising from π–π stacking. The upper corresponds to the stacking across (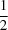, 1, 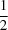), while the lower corresponds to the stacking across (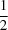, 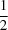, 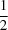).

**Figure 5:**
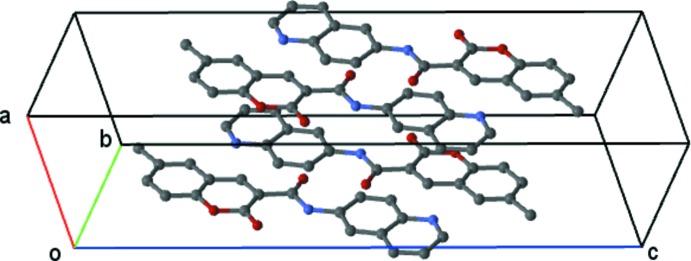
View of the π–π stacking along the *b* axis.

In this structure, C/N⋯C contacts prevail over the C—H⋯C ones. In fact, the packing in (1) is built up by several π–π inter­actions (Table 4 ▸). The red spots in the frontal zone of the surface correspond to these close contacts. Furthermore, the FP plot also reveals an intense cluster at *d*_e_/*d*_i_ at 1.8 Å characteristic of C⋯C contacts. Also, when the surface is mapped with shape index, several complementary triangular red hollows and blue bumps appear that are characteristic of the six-ring stacking (Fig. 4 ▸). The mol­ecules stack in a column in a head-to-tail fashion along the *b* axis (Fig. 5 ▸). The mol­ecules in these stacks lie across centres of symmetry at (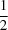, 1, 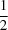), a centrosymetrically related contact between the pyran and pyridine rings, and across the centre at (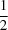, 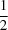, 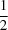), which involves three short centrosymmetrically related contacts: (i) between the pyran and pyridine rings, (ii) between the pyran ring and the quinoline phenyl ring and (iii) between the coumarin phenyl ring and the pyridine ring.

## Database survey   

As reported by Gomes *et al.* (2016 ▸), a search made in the Cambridge Structural Database (CSD, Version 35.7; Groom *et al.*, 2016 ▸) revealed the existence of 35 deposited compounds (42 mol­ecules) containing the coumarin carboxamide unit, all of which contained the same intra­molecular hydrogen bonds. The present compound also contains these bonds, as described above.

## Synthesis and crystallization   

6-Methyl­coumarin-3-carb­oxy­lic acid (Murata *et al.*., 2005 ▸) (1 mmol) was dissolved in di­chloro­methane and 3-[3-(di­methyl­amino)­prop­yl]-1-ethyl­carbodi­imide (1.10 mmol) and 4-di­methyl­amino­pyridine (1.10 mmol) were added. The mixture was kept under a flux of argon at 273 K for 5 min. 6-Amino­quinoline (1 mmol) was then added in small portions. The reaction mixture was stirred for 4 h at room temperature. The obtained precipitate was filtered off and recrystallized from methanol to give colourless needles of (1). Overall yield: 53%; m.p. 545–546 K.

## Refinement   

H atoms were treated as riding atoms, with aromatic C—H = 0.95 Å, with *U*_iso_(H) = 1.2*U*_eq_(C), and methyl C—H = 0.98 Å, with *U*_iso_(H) = 1.5*U*_eq_(C). The amino H atoms were freely refined. Crystal data, data collection and structure refinement details are summarized in Table 5 ▸.

## Supplementary Material

Crystal structure: contains datablock(s) 1, general. DOI: 10.1107/S2056989016011026/hb7596sup1.cif

Structure factors: contains datablock(s) I. DOI: 10.1107/S2056989016011026/hb7596Isup2.hkl

CCDC reference: 1491340

Additional supporting information: crystallographic information; 3D view; checkCIF report

## Figures and Tables

**Table 1:** Hydrogen-bond geometry (Å, °)

*D*—H⋯*A*	*D*—H	H⋯*A*	*D*⋯*A*	*D*—H⋯*A*
C314—H314⋯O31^i^	0.95	2.50	3.278 (2)	139
C8—H8⋯N311^ii^	0.95	2.68	3.394 (3)	133
C317—H317⋯O31	0.95	2.29	2.903 (2)	122
N32—H32⋯O2	0.907 (18)	1.879 (18)	2.686 (2)	147.3 (15)

Symmetry codes: (i) 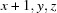; (ii) 
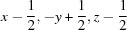
; (iii) 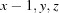.

**Table 2:** Selected dihedral angles (°)

Compound	θ_1_ (°)	θ_2_ (°)	θ_3_ (°)
(1)	6.08 (6)	5.0 (12)	1.73 (11)

Notes: θ_1_ is the dihedral angle between the mean planes of the coumarin and quinoline rings; θ_2_ is the dihedral angle between the mean plane of the coumarin ring and the plane defined by atoms O31/C31/N32; θ_3_ is the dihedral angle between the mean plane of the quinoline ring and the plane defined by atoms O31/C31/N32.

**Table 3:** Percentages of atom–atom contacts for (1) (%)

Contact	H⋯H	H⋯O/O⋯H	H⋯N/N⋯H	C⋯C	N⋯C/C⋯N	H⋯C/C⋯H
(%)	40.6	21.2	3.3	13.2	4.7	14.3

**Table 4:** Selected π–π contacts

Compound	*CgI*	*CgJ*(aru)	*Cg*–*Cg* (Å)	*CgI*_Perp (Å)	*CgJ*_Perp (Å)	Slippage (Å)
1	*Cg*1	*Cg*2(−*x* + 1, −*y*, −*z* − 1)	3.548 (2)	3.1477 (4)	3.3051 (4)	1.290
1	*Cg*1	*Cg*2(−*x* + 1, −*y* + 1, −*z* − 1)	3.911 (3)	−3.3848 (4)	−3.3352 (4)	2.043
1	*Cg*1	*Cg*4(−*x* + 1, −*y* + 1, −*z* − 1)	3.525 (2)	−3.3851 (4)	−3.2952 (4)	1.252
1	*Cg*2	*Cg*1(−*x* + 1, −*y*, −*z* − 1)	3.548 (2)	3.3050 (4)	3.1476 (4)	1.637
1	*Cg*2	*Cg*1(−*x* + 1, −*y* + 1, −*z* − 1)	3.911 (3)	−3.3352 (4)	−3.3849 (4)	1.960
1	*Cg*2	*Cg*3(−*x* + 1, −*y* + 1, −*z* − 1)	3.797 (3)	−3.3389 (4)	−3.5276 (5)	1.406
1	*Cg*3	*Cg*2(−*x* + 1, −*y* + 1, −*z* − 1)	3.798 (3)	−3.5277 (5)	−3.3388 (4)	1.809
1	*Cg*4	*Cg*1(−*x* + 1, −*y* + 1, −*z* − 1)	3.525 (2)	−3.2951 (4)	−3.3852 (4)	0.983

Notes: *CgI*(*J*) = Plane number *I*(*J*); *Cg*–*Cg* = distance between ring centroids; *CgI*_Perp = perpendicular distance of *CgI* on ring *J*; *CgJ*_Perp = perpendicular distance of *CgJ* on ring *I*; Slippage = distance between *CgI* and perpendicular projection of *CgJ* on ring *I*.

Plane 1 is the plane of the coumarin pyran ring with *Cg*1 as centroid; Plane 2 is the plane of the quinoline pyridine ring with *Cg*2 as centroid; Plane 3 is the plane of the coumarin phenyl ring with *Cg*3 as centroid; Plane 4 is the plane of the quinoline phenyl ring with *Cg*4 as centroid.

Some planes are repeated since they are inclined to each other and as a result give slightly different slippages

**Table 5:** Experimental details

Crystal data	
Chemical formula	C_20_H_14_N_2_O_3_
*M*_r_	330.33
Crystal system, space group	Monoclinic, *P*2_1_/*n*
Temperature (K)	100
*a*, *b*, *c* (Å)	7.799 (3), 7.014 (3), 27.640 (18)
β (°)	90.18 (6)
*V* (Å^3^)	1512.0 (13)
*Z*	4
Radiation type	Synchrotron, λ = 0.68891 Å
μ (mm^−1^)	0.09
Crystal size (mm)	0.18 × 0.01 × 0.004

Data collection	
Diffractometer	Three-circle diffractometer
Absorption correction	Empirical (using intensity measurements) (aimless *CCP4*; Evans, 2006 ▸)
No. of measured, independent and observed [*I* > 2σ(*I*)] reflections	18408, 4587, 3717
*R*_int_	0.060
(sin θ/λ)_max_ (Å^−1^)	0.714

Refinement	
*R*[*F* ^2^ > 2σ(*F* ^2^)], *wR*(*F* ^2^), *S*	0.051, 0.156, 1.13
No. of reflections	4587
No. of parameters	231
H-atom treatment	H atoms treated by a mixture of independent and constrained refinement
Δρ_max_, Δρ_min_ (e Å^−3^)	0.54, −0.25

Computer programs: GDA http://www.opengda.org/OpenGDA.html, XIA2 0.4.0.370-g47f3bc3 (Winter, 2010 ▸), *SHELXT* (Sheldrick, 2015*a* ▸), ShelXle (Hübschle *et al.*, 2011 ▸), *SHELXL2014* (Sheldrick, 2015*b* ▸), *Mercury* (Macrae *et al.*, 2006 ▸) and *PLATON* (Spek, 2009 ▸).

## supplementary crystallographic information

### Crystal data

**Table:**

C_20_H_14_N_2_O_3_	*F*(000) = 688
*M**_r_* = 330.33	*D*_x_ = 1.451 Mg m^−^^3^
Monoclinic, *P*2_1_/*n*	Synchrotron' radiation, λ = 0.68891 Å
*a* = 7.799 (3) Å	Cell parameters from 3773 reflections
*b* = 7.014 (3) Å	θ = 2.6–33.9°
*c* = 27.640 (18) Å	µ = 0.09 mm^−^^1^
β = 90.18 (6)°	*T* = 100 K
*V* = 1512.0 (13) Å^3^	Needle, colourless
*Z* = 4	0.18 × 0.01 × 0.004 mm

### Data collection

**Table:**

Three-circle diffractometer	4587 independent reflections
Radiation source: synchrotron, DLS beamline I19, undulator	3717 reflections with *I* > 2σ(*I*)
Si 111, double crystal monochromator	*R*_int_ = 0.060
Detector resolution: 5.81 pixels mm^-1^	θ_max_ = 29.5°, θ_min_ = 2.9°
profile data from ω–scans	*h* = −11→11
Absorption correction: empirical (using intensity measurements) aimless ccp4 (Evans, 2006)	*k* = −10→10
	*l* = −39→39
18408 measured reflections	

### Refinement

**Table:**

Refinement on *F*^2^	0 restraints
Least-squares matrix: full	Hydrogen site location: mixed
*R*[*F*^2^ > 2σ(*F*^2^)] = 0.051	H atoms treated by a mixture of independent and constrained refinement
*wR*(*F*^2^) = 0.156	*w* = 1/[σ^2^(*F*_o_^2^) + (0.0954*P*)^2^] where *P* = (*F*_o_^2^ + 2*F*_c_^2^)/3
*S* = 1.13	(Δ/σ)_max_ = 0.001
4587 reflections	Δρ_max_ = 0.54 e Å^−^^3^
231 parameters	Δρ_min_ = −0.25 e Å^−^^3^

### Special details

**Table:**

Geometry. All esds (except the esd in the dihedral angle between two l.s. planes) are estimated using the full covariance matrix. The cell esds are taken into account individually in the estimation of esds in distances, angles and torsion angles; correlations between esds in cell parameters are only used when they are defined by crystal symmetry. An approximate (isotropic) treatment of cell esds is used for estimating esds involving l.s. planes.

### Fractional atomic coordinates and isotropic or equivalent isotropic displacement parameters (Å^2^)

**Table:**

	*x*	*y*	*z*	*U*_iso_*/*U*_eq_	
O1	0.61633 (10)	0.57511 (12)	0.33247 (3)	0.02193 (19)	
O2	0.42390 (10)	0.67655 (12)	0.38475 (3)	0.0244 (2)	
O31	0.79011 (10)	0.65810 (12)	0.49728 (3)	0.0240 (2)	
N32	0.50968 (12)	0.71842 (13)	0.47835 (3)	0.0186 (2)	
N311	0.21868 (12)	0.98576 (13)	0.65151 (3)	0.0204 (2)	
C2	0.57274 (14)	0.62613 (16)	0.37867 (4)	0.0196 (2)	
C3	0.70619 (13)	0.61438 (15)	0.41571 (4)	0.0177 (2)	
C4	0.86585 (13)	0.55411 (15)	0.40316 (4)	0.0180 (2)	
H4	0.9519	0.5451	0.4275	0.022*	
C5	1.07250 (14)	0.44450 (15)	0.33910 (4)	0.0200 (2)	
H5	1.1628	0.4362	0.3622	0.024*	
C4A	0.90818 (14)	0.50369 (15)	0.35425 (4)	0.0182 (2)	
C6	1.10480 (15)	0.39806 (16)	0.29103 (4)	0.0222 (2)	
C7	0.97020 (15)	0.41362 (17)	0.25763 (4)	0.0238 (2)	
H7	0.9904	0.3821	0.2247	0.029*	
C8	0.80789 (15)	0.47398 (17)	0.27134 (4)	0.0230 (2)	
H8	0.7184	0.4858	0.2482	0.028*	
C8A	0.77937 (14)	0.51669 (16)	0.31972 (4)	0.0195 (2)	
C31	0.67345 (13)	0.66578 (15)	0.46783 (4)	0.0185 (2)	
C34A	0.18814 (13)	0.89163 (15)	0.56639 (4)	0.0176 (2)	
C38A	0.28705 (13)	0.91692 (15)	0.60890 (4)	0.0178 (2)	
C61	1.27981 (16)	0.3329 (2)	0.27477 (5)	0.0312 (3)	
H61A	1.3103	0.2147	0.2916	0.047*	
H61B	1.3647	0.4316	0.2824	0.047*	
H61C	1.2781	0.3101	0.2398	0.047*	
C312	0.05500 (14)	1.03063 (16)	0.65149 (4)	0.0219 (2)	
H312	0.0075	1.0795	0.6806	0.026*	
C313	−0.05445 (14)	1.01098 (16)	0.61122 (4)	0.0219 (2)	
H313	−0.1717	1.0466	0.6133	0.026*	
C314	0.01123 (13)	0.93934 (16)	0.56876 (4)	0.0199 (2)	
H314	−0.0609	0.9220	0.5413	0.024*	
C315	0.26757 (13)	0.82186 (15)	0.52375 (4)	0.0183 (2)	
H315	0.2006	0.8037	0.4954	0.022*	
C316	0.44016 (14)	0.78004 (15)	0.52286 (4)	0.0178 (2)	
C317	0.53856 (14)	0.80040 (16)	0.56555 (4)	0.0197 (2)	
H317	0.6569	0.7683	0.5655	0.024*	
C318	0.46224 (14)	0.86712 (16)	0.60735 (4)	0.0198 (2)	
H318	0.5297	0.8798	0.6358	0.024*	
H32	0.440 (2)	0.709 (3)	0.4521 (6)	0.042 (5)*	

### Atomic displacement parameters (Å^2^)

**Table:**

	*U*^11^	*U*^22^	*U*^33^	*U*^12^	*U*^13^	*U*^23^
O1	0.0188 (4)	0.0271 (4)	0.0199 (4)	−0.0001 (3)	0.0003 (3)	−0.0025 (3)
O2	0.0186 (4)	0.0295 (5)	0.0250 (4)	0.0018 (3)	−0.0007 (3)	−0.0027 (3)
O31	0.0209 (4)	0.0295 (5)	0.0216 (4)	0.0024 (3)	−0.0005 (3)	−0.0043 (3)
N32	0.0184 (4)	0.0194 (5)	0.0180 (4)	0.0012 (3)	0.0018 (3)	−0.0009 (3)
N311	0.0226 (4)	0.0201 (5)	0.0186 (5)	−0.0011 (3)	0.0019 (3)	−0.0011 (3)
C2	0.0200 (5)	0.0186 (5)	0.0203 (5)	−0.0020 (4)	0.0014 (4)	−0.0007 (4)
C3	0.0185 (5)	0.0164 (5)	0.0183 (5)	−0.0012 (4)	0.0012 (4)	−0.0004 (4)
C4	0.0192 (5)	0.0155 (5)	0.0193 (5)	−0.0012 (4)	0.0008 (4)	0.0000 (4)
C5	0.0208 (5)	0.0178 (5)	0.0214 (5)	−0.0001 (4)	0.0019 (4)	−0.0001 (4)
C4A	0.0199 (5)	0.0154 (5)	0.0194 (5)	−0.0022 (4)	0.0021 (4)	−0.0006 (4)
C6	0.0242 (5)	0.0197 (5)	0.0227 (5)	−0.0012 (4)	0.0046 (4)	−0.0018 (4)
C7	0.0273 (5)	0.0240 (6)	0.0202 (5)	−0.0030 (4)	0.0042 (4)	−0.0024 (4)
C8	0.0248 (5)	0.0258 (6)	0.0185 (5)	−0.0030 (4)	−0.0011 (4)	−0.0013 (4)
C8A	0.0188 (5)	0.0193 (5)	0.0202 (5)	−0.0024 (4)	0.0021 (4)	−0.0007 (4)
C31	0.0194 (5)	0.0151 (5)	0.0212 (5)	−0.0009 (4)	0.0025 (4)	−0.0005 (4)
C34A	0.0178 (5)	0.0144 (5)	0.0205 (5)	−0.0002 (3)	0.0016 (4)	0.0011 (4)
C38A	0.0194 (5)	0.0158 (5)	0.0181 (5)	−0.0013 (4)	0.0016 (4)	0.0003 (4)
C61	0.0253 (6)	0.0408 (8)	0.0274 (6)	0.0069 (5)	0.0052 (5)	−0.0060 (5)
C312	0.0238 (5)	0.0204 (5)	0.0214 (5)	−0.0012 (4)	0.0057 (4)	−0.0012 (4)
C313	0.0188 (5)	0.0216 (5)	0.0251 (6)	0.0002 (4)	0.0032 (4)	0.0006 (4)
C314	0.0180 (5)	0.0202 (5)	0.0215 (5)	−0.0006 (4)	−0.0001 (4)	0.0015 (4)
C315	0.0195 (5)	0.0167 (5)	0.0188 (5)	0.0004 (4)	−0.0001 (4)	0.0003 (4)
C316	0.0201 (5)	0.0148 (5)	0.0185 (5)	−0.0001 (4)	0.0029 (4)	0.0006 (4)
C317	0.0183 (5)	0.0203 (5)	0.0206 (5)	0.0010 (4)	0.0016 (4)	0.0002 (4)
C318	0.0204 (5)	0.0207 (5)	0.0185 (5)	−0.0005 (4)	−0.0011 (4)	−0.0002 (4)

### Geometric parameters (Å, º)

**Table:**

O1—C2	1.3701 (16)	C7—H7	0.9500
O1—C8A	1.3827 (14)	C8—C8A	1.3887 (18)
O2—C2	1.2255 (14)	C8—H8	0.9500
O31—C31	1.2201 (16)	C34A—C38A	1.4149 (18)
N32—C31	1.3618 (14)	C34A—C315	1.4200 (17)
N32—C316	1.4136 (16)	C34A—C314	1.4214 (15)
N32—H32	0.907 (18)	C38A—C318	1.4111 (16)
N311—C312	1.3147 (15)	C61—H61A	0.9800
N311—C38A	1.3814 (16)	C61—H61B	0.9800
C2—C3	1.4600 (18)	C61—H61C	0.9800
C3—C4	1.3609 (15)	C312—C313	1.4075 (19)
C3—C31	1.5075 (18)	C312—H312	0.9500
C4—C4A	1.4368 (17)	C313—C314	1.3769 (17)
C4—H4	0.9500	C313—H313	0.9500
C5—C6	1.3918 (18)	C314—H314	0.9500
C5—C4A	1.4119 (16)	C315—C316	1.3779 (15)
C5—H5	0.9500	C315—H315	0.9500
C4A—C8A	1.3866 (18)	C316—C317	1.4128 (18)
C6—C7	1.4000 (19)	C317—C318	1.3830 (17)
C6—C61	1.5092 (17)	C317—H317	0.9500
C7—C8	1.3886 (17)	C318—H318	0.9500
			
C2—O1—C8A	123.10 (10)	N32—C31—C3	115.51 (11)
C31—N32—C316	129.15 (11)	C38A—C34A—C315	119.63 (10)
C31—N32—H32	111.5 (11)	C38A—C34A—C314	117.32 (10)
C316—N32—H32	119.3 (11)	C315—C34A—C314	123.05 (11)
C312—N311—C38A	117.43 (11)	N311—C38A—C318	119.26 (11)
O2—C2—O1	116.17 (11)	N311—C38A—C34A	122.75 (10)
O2—C2—C3	126.42 (11)	C318—C38A—C34A	117.98 (10)
O1—C2—C3	117.42 (10)	C6—C61—H61A	109.5
C4—C3—C2	119.32 (11)	C6—C61—H61B	109.5
C4—C3—C31	118.40 (11)	H61A—C61—H61B	109.5
C2—C3—C31	122.28 (10)	C6—C61—H61C	109.5
C3—C4—C4A	121.96 (11)	H61A—C61—H61C	109.5
C3—C4—H4	119.0	H61B—C61—H61C	109.5
C4A—C4—H4	119.0	N311—C312—C313	124.28 (11)
C6—C5—C4A	121.27 (12)	N311—C312—H312	117.9
C6—C5—H5	119.4	C313—C312—H312	117.9
C4A—C5—H5	119.4	C314—C313—C312	118.92 (10)
C8A—C4A—C5	118.13 (11)	C314—C313—H313	120.5
C8A—C4A—C4	117.61 (11)	C312—C313—H313	120.5
C5—C4A—C4	124.25 (11)	C313—C314—C34A	119.27 (11)
C5—C6—C7	118.28 (11)	C313—C314—H314	120.4
C5—C6—C61	121.45 (12)	C34A—C314—H314	120.4
C7—C6—C61	120.28 (11)	C316—C315—C34A	121.13 (11)
C8—C7—C6	121.73 (11)	C316—C315—H315	119.4
C8—C7—H7	119.1	C34A—C315—H315	119.4
C6—C7—H7	119.1	C315—C316—C317	119.45 (11)
C7—C8—C8A	118.52 (12)	C315—C316—N32	117.26 (11)
C7—C8—H8	120.7	C317—C316—N32	123.29 (10)
C8A—C8—H8	120.7	C318—C317—C316	119.86 (10)
O1—C8A—C4A	120.59 (10)	C318—C317—H317	120.1
O1—C8A—C8	117.36 (11)	C316—C317—H317	120.1
C4A—C8A—C8	122.06 (11)	C317—C318—C38A	121.90 (11)
O31—C31—N32	124.57 (11)	C317—C318—H318	119.0
O31—C31—C3	119.91 (10)	C38A—C318—H318	119.0
			
C8A—O1—C2—O2	179.41 (9)	C4—C3—C31—O31	2.12 (16)
C8A—O1—C2—C3	−0.94 (15)	C2—C3—C31—O31	−178.23 (10)
O2—C2—C3—C4	179.54 (11)	C4—C3—C31—N32	−177.74 (9)
O1—C2—C3—C4	−0.06 (15)	C2—C3—C31—N32	1.91 (15)
O2—C2—C3—C31	−0.09 (18)	C312—N311—C38A—C318	179.98 (10)
O1—C2—C3—C31	−179.70 (9)	C312—N311—C38A—C34A	−0.77 (16)
C2—C3—C4—C4A	0.81 (16)	C315—C34A—C38A—N311	179.40 (10)
C31—C3—C4—C4A	−179.54 (9)	C314—C34A—C38A—N311	−0.32 (16)
C6—C5—C4A—C8A	−0.76 (16)	C315—C34A—C38A—C318	−1.34 (15)
C6—C5—C4A—C4	−179.86 (10)	C314—C34A—C38A—C318	178.94 (10)
C3—C4—C4A—C8A	−0.58 (16)	C38A—N311—C312—C313	0.74 (17)
C3—C4—C4A—C5	178.52 (10)	N311—C312—C313—C314	0.39 (18)
C4A—C5—C6—C7	0.76 (17)	C312—C313—C314—C34A	−1.51 (16)
C4A—C5—C6—C61	−179.40 (11)	C38A—C34A—C314—C313	1.45 (15)
C5—C6—C7—C8	0.15 (18)	C315—C34A—C314—C313	−178.25 (10)
C61—C6—C7—C8	−179.69 (11)	C38A—C34A—C315—C316	−0.68 (16)
C6—C7—C8—C8A	−1.03 (18)	C314—C34A—C315—C316	179.02 (10)
C2—O1—C8A—C4A	1.19 (16)	C34A—C315—C316—C317	2.29 (16)
C2—O1—C8A—C8	−178.25 (10)	C34A—C315—C316—N32	−177.58 (9)
C5—C4A—C8A—O1	−179.57 (9)	C31—N32—C316—C315	−178.87 (10)
C4—C4A—C8A—O1	−0.41 (15)	C31—N32—C316—C317	1.27 (18)
C5—C4A—C8A—C8	−0.15 (17)	C315—C316—C317—C318	−1.86 (16)
C4—C4A—C8A—C8	179.00 (10)	N32—C316—C317—C318	178.01 (10)
C7—C8—C8A—O1	−179.54 (10)	C316—C317—C318—C38A	−0.20 (17)
C7—C8—C8A—C4A	1.03 (18)	N311—C38A—C318—C317	−178.93 (10)
C316—N32—C31—O31	2.43 (19)	C34A—C38A—C318—C317	1.78 (16)
C316—N32—C31—C3	−177.72 (10)		

### Hydrogen-bond geometry (Å, º)

**Table:**

*D*—H···*A*	*D*—H	H···*A*	*D*···*A*	*D*—H···*A*
C314—H314···O31^i^	0.95	2.50	3.278 (2)	139
C8—H8···N311^ii^	0.95	2.68	3.394 (3)	133
C317—H317···O31	0.95	2.29	2.903 (2)	122
N32—H32···O2	0.907 (18)	1.879 (18)	2.686 (2)	147.3 (15)

Symmetry codes: (i) *x*−1, *y*, *z*; (ii) *x*+1/2, −*y*+3/2, *z*−1/2.
